# Increased Regeneration Following Stress-Induced Lung Injury in Bleomycin-Treated Chimeric Mice with CD44 Knockout Mesenchymal Cells

**DOI:** 10.3390/cells8101211

**Published:** 2019-10-07

**Authors:** Dmytro Petukhov, Mark Richter-Dayan, Zvi Fridlender, Raphael Breuer, Shulamit B. Wallach-Dayan

**Affiliations:** 1 Lung Cellular and Molecular Biology Laboratory, Institute of Pulmonary Medicine, Hadassah–Hebrew University Medical Center, PO Box 12000, Qiryat Hadassah, Jerusalem 91120, Israel; petukhov@hadassah.org.il (D.P.); fridlender@hadassah.org.il (Z.F.); raffibreuer@gmail.com (R.B.); 2 Department of Emergency Medicine, Hadassah–Hebrew University Medical Center, PO Box 12000, Qiryat Hadassah, Jerusalem 91120, Israel; richter@hadassah.org.il; 3 Department of Pathology, Boston University School of Medicine, 72 East Concord St., Boston, MA 02118, USA

**Keywords:** regeneration, fibrosis, oxidative stress, bleomycin, cell-survival, epithelial-mesenchymal transition, ATM, CD44

## Abstract

CD44, an adhesion-molecule promoting cell-migration, is shown here to increase in stress conditions following bleomycin-induced apoptosis in alveolar epithelial cells (AECs), a main target of lung injury. In vivo, it inhibits tissue regeneration and leads to fibrosis. We show that some AECs survive by the ataxia-telangiectasia mutated kinase/ATM pathway, and undergo a CD44-mediated epithelial-mesenchymal transdifferentiation (EMT) with migratory capacities in vitro, and in vivo. We assessed apoptosis vs. proliferation of AECs following bleomycin, ATM/P53 signaling pathway in AECs, and CD44 involvement in EMT, cell motility and tissue regeneration in vitro and in vivo. Expression of survival genes, CD44, and ATM/p53 pathway was elevated in AECs surviving bleomycin injury, as were the markers of EMT (downregulation of E-cadherin, upregulation of N-cadherin and vimentin, nuclear translocation of β-catenin). Inhibition of CD44 decreased AECs transdifferentiation. Bleomycin-treated chimeric CD44^KO^-mice had decreased EMT markers, ATM, and mesenchymal cells (α-SMA^+^) accumulation in lung, increased surfactant-b, diminished lung mesenchymal cell motility, and increased lung tissue regenerative capacity following bleomycin injury, as indicated by lung collagen content and semiquantitave morphological index scoring. Thus, AECs surviving lung injury are plastic and undergo ATM-mediated, CD44-dependent transdifferentiation, preventing tissue regeneration and promoting fibrosis. Synthetic or natural compounds that downregulate CD44 may improve tissue regeneration following injury.

## 1. Introduction

We previously detected bleomycin-induced reactive oxygen species (ROS) resulting in oxidative stress, mitochondrial leakage, DNA damage response (DDR), and apoptosis, in normal alveolar epithelial cells (AECs) [1]. Interestingly, the main inducers of apoptosis can also promote EMT, suggesting that EMT could potentially serve as a pathway for escape from cell death [2]. EMT occurs during embryonic development and organogenesis and represents an obstacle to tissue regeneration following injury. Thus, EMT may also cause diseases such as fibrosis, cancer, rheumatoid arthritis, tissue degradation, and others [3,4]. Many EMT inducers act directly or indirectly by repressing E-cadherin, increasing vimentin and α–smooth muscle actin (αSMA) [3], a set of incidences that promote accumulation of myofibroblasts in injured tissues, which characterizes fibrotic diseases [5], including idiopathic pulmonary fibrosis (IPF). CD44 is described as one of the primary factors contributing to EMT, especially in cancer [4], where it is regarded as one of the stem markers, but also in inflammation [6], atrial fibrosis [7], and renal fibrosis [8]. Involvement of CD44 in EMT during pulmonary fibrosis as opposed to tissue regeneration (both in clinic and in experimental animals) is more scant [9]. Moreover, IPF in aging, which as opposed to juvenile subjects, lack the possibility of tissue regeneration and do not resolve fibrosis [10], correlates with increments in oxidative stress [11].

In response to DDR [1], cells activate factors halting cell cycle while a decision is made regarding repair and survival, or death [12]. ATM, which initiates DDR, triggers cell cycle checkpoints, and manages DNA repair with phosphorylation of downstream targets, including p53, CHK2, and BRCA1 [13], and activation of NF-κB and Erk, involved with survival signaling and proliferation, in particular after cell stress and radiation [14]. Under oxidative stress, ATM has been shown to promote the translocation of β-catenin into the nucleus where it activates target genes to promote cell growth and EMT [15]. Further promotion of cell survival and migration is through the p53-mediated expression of CD44 [16]. In this study, we determined the association between stress response and ATM/Chk1 proliferation cascades, with expression of CD44, in AECs with upregulation of mesenchymal cell markers and β-catenin translocation and a migratory phenotype following tissue injury with bleomycin. We also addressed the question about whether CD44, specifically expressed in mesenchymal cells, in bleomycin-treated chimeric-CD44^KO^ mice, is essential in lung EMT, suppression of regeneration and evolution of fibrosis. In aging, this suppression of regeneration may mean permanent tissue damage; in turn, taking over the control over this damage may allow one to address the standing issues of regeneration and rejuvenation in senescence.

## 2. Materials and Methods

### 2.1. MLE Cell-line and Culture

Murine type-II lung epithelial cell-line (ATCC, MLE-12), an extensively used model, cultured in 5% CO2 37 °C in a humidified atmosphere with HITES medium, with 2 mM l-glutamine, 10% heat-inactivated FBS, and 5 mM Pen-Strep (Bilogical Industries, Beit-HaEmek, Israel) as we detailed [1]. MLEs (0.5 × 10^6^) were cultured in HITES medium, with or without 200 mU/mL of bleomycin and with or without anti-CD44 mAb, clone-KM81 and IgG control, clone-4D2 (OriGene Technologies GmbH, Herford, Germany), which were also used for flow cytometry.

### 2.2. Mice and Chimerism

Male mice 11–12-week-old, C57BL/6J WT (Harlan Ltd., Kiryat Weizmann Israel), and CD44 knockout (CD44^KO^) [17,18]. Chimerism was obtained by adoptive transfer of WT splenocytes into CD44^KO^ as detailed previously [19]. Mice were maintained under specific pathogen-free conditions in the Animal Unit of the Hadassah–Hebrew University Medical Center and experimental procedures approved by the Institutional Animal Care and Use Committee (Res. No. MD-15-14590-5, Appr. Date 16.12.2015).

### 2.3. Assessment of Regenerative Capacity

Experimental fibrosis vs. tissue regeneration was induced in mice with bleomycin (Asta-Medica AB, Taby, Sweden) by oropharyngeal aspiration (OA-BLM), sacrificed by a lethal dose of pentothal (CTS, Kiryat Malachi, Israel) at different times following OA-BLM as we detailed [20]. Left lung tissue sections (paraffin-embedded) for examination of regenerative capacity or fibrosis, bronchoalveolar lavage (BAL), and immunohistochemistry (IHC), hematoxylin and eosin (H&E) deparaffinized sections [19]. Semiquantitave morphological index (SMI), scoring lack of regeneration (fibrosis) in lung sections was used, as previously described [21].

The lower right lobe served for fibroblast isolation and the upper right lobe, 10 mg of homogenized tissue, for soluble Sircol collagen (Biocolor kit) evaluated at 540 nm with a spectrophotometer [22].

### 2.4. Annexin-V & BrdU Uptake in Flow Cytometry

As we described previously, we sorted out by FACS cell sorter [19], annexin-V-negative, bleomycin-treated MLEs [1] and quantified proliferation by BrdU uptake [23]. Briefly, FITC-conjugated annexin-V (BD Pharmingen, Franklin Lakes, NJ, USA), was added to 0.5 × 10^6^ MLE adherent cells in the culture flask (1 μg, 30 min). For proliferation, BrdU (SigmaAldrich, Darmstadt, Germany), was added to MLEs (6 h, 20 μM). Cells were pelleted (400 *g*, 5 min) and resuspended in FACS buffer. BrdU was detected by staining with FITC-conjugated anti-BrdU (BD Pharmingen, Franklin Lakes, NJ, USA), (30 min, RT). Propidium iodide (PI), (Calbiochem, La Jolla, CA, USA) was added (5 μg/mL, 15 min, on ice). Flow cytometry analysis by plotting green fluorescence (FL1)/FITC-annexin-V vs. red fluorescence (FL2)/PI-positive cells and proliferation distribution by measuring corresponding BrdU uptake versus total DNA content (cells in S phase) [23], were performed with a FACStar (Becton Dickinson, Mountain View, CA, USA).

### 2.5. EMT and ATM in IHC

Sections were incubated with anti-ATM (Invitrogen, Rockford, IL, USA), anti-αSMA (DAKO A/S, Glostrup, Denmark) or anti–surfactant-B Ab (Merck, Darmstadt, Germany), reincubated with biotinylated anti-mouse, or anti-rabbit Ab, streptavidin–biotin complex, peroxidase substrate (N-Histofine kit, Nichirei Biosciences, Tokyo, Japan) and counterstained with hematoxylin [19].

### 2.6. Lung Mesenchymal Cell Isolation and Culture

Lungs were minced, incubated (45 min, 37 °C, 5% CO_2_) in collagenase 1 mg/mL-PBS (Sigma-Aldrich, St. Louis, MO, USA), passed through a cell dissociation sieve (Sigma-Aldrich, St. Louis, MO, USA), subjected to fibroblast culture medium and verified as in flow cytometry as we previously detailed [19].

### 2.7. Motility

Cells were cultured in medium containing 0.5% BSA and 20% FCS, up to 50–70% confluence, scratched with a pipette tip and washed with PBS. The “wounded” areas were cultured in starvation (0.5% FCS) for additional 24 h. Images of regeneration and migration taken under phase contrast microscope were analyzed by TIMNA software.

### 2.8. Immunofluorescence (IF)

Confocal microscopy procedures were as in our previous work [24]. MLEs were cultured on 0.2-mm-thick coverslips or pelleted and incubated with 1:100 β-catenin, anti–vimentin, E-cadherin, N-cadherin and Cy5-conjugated mAbs (R&D Biotechnologies, Minneapolis, MN, USA). These were also used for Wb. Monolayers were analyzed by confocal microscopy (Axio Scope 2; Carl Zeiss AG, Oberkochen, Germany).

### 2.9. Immunoblot (IB)

Cell lysates were obtained separated by 10% sodium dodecyl sulfate poly-acrylamide gel electrophoresis under reducing conditions as previously described [25]. After electrophoresis, samples were transferred to a nitrocellulose membrane (Bio-Rad, Hercules, CA, USA) [25]. Membranes were incubated in 5% dry milk TBS-T buffer for 1hr, with vimentin, E-cadherin or N-cadherin (1:1000) mAbs (R&D Biotechnologies, Minneapolis, MN, USA). Phospho^(Ser1981)^-ATM mAb (10H11) (Thermo-Fisher Scientific, Waltham, MA, USA) and ATM mAb, (Invitrogen, Rockford, IL, USA) were used to detect ATM expression and activity. The membranes were washed with TBS-T and incubated with horseradish peroxidase (HRP)-conjugated anti-rabbit IgG (1:1000) (Jackson Immunoresearch, West Grove, PA, USA) in 5% dry milk and TBS-T for 1 h. Thereafter, blots were developed with the EZ-ECL chemiluminescence detection kit (Bilogical Industries, Beit-HaEmek, Israel) according to the manufacturer’s instructions. β-actin (1:500) (Santa Cruz Biotechnology, Dallas, TX, USA) was used for loading control [25].

ATM activity was assayed via IB of the phosphorylation products, as described using Phospho-ATM vs. ATM mAbs (1:500) [26].

### 2.10. Phosphorylated Proteins

An antibody array (Hypomatrix, Worcester, MA, USA) containing specific antibodies detecting of a range of signaling-molecules and kinases was used to study phosphorylation in bleomycin-treated MLEs as we described [25]. Briefly, the array membrane was incubated (2hr, RT) with lysates from BLM- annexin-V^(−)^ MLEs, washed with PBS and blotted (2 h, RT) with HRP-conjugated anti-Phosphotyrosine RC20 (2 mg/mL), (BD Transduction Laboratories, Franklin Lakes, NJ, USA), followed by ECL-detection [25,26].

### 2.11. Survival Genes

A mouse apoptosis pathway gene array kit (GEArray; SuperArray Inc., Bethesda, MD, USA) was used to determine the expression levels of multiple genes involved in epithelial cell apoptosis as we previously detailed [19]. Briefly, cDNA probes were denatured and hybridized with the SuperArray membrane, which was then scanned by the X-ray. A pool of four cDNA spots for each gene was used and digitized using ScanAlyze software (shareware, available at http://eisenlab.org/software.html), and normalized by subtraction of the background average intensity value of three spots containing plasmid DNA (PUC18). The average of two RPL13A spots was used as a positive control and set as the baseline value compared signal to other spots intensity using the GEarray analyzer program.

### 2.12. Data Analysis and Representation

The nonparametric Kruskall-Wallis test was applied to compare variables measured at different time intervals or following different treatments. Multiple pairwise comparisons were performed using the Mann-Whitney non-parametric test with the Bonferroni correction for significance. Two-way ANOVA was used to assess time and treatment effects. All statistical tests were two-tailed, and a p-value of 0.05 or less was considered significant.

## 3. Results

### 3.1. A Portion of AECs, Mouse Lung Epithelial (MLE) Cells, Exposed to Bleomycin Resist Apoptosis and Upregulate Cell-Survival Genes

**Figure 1:**
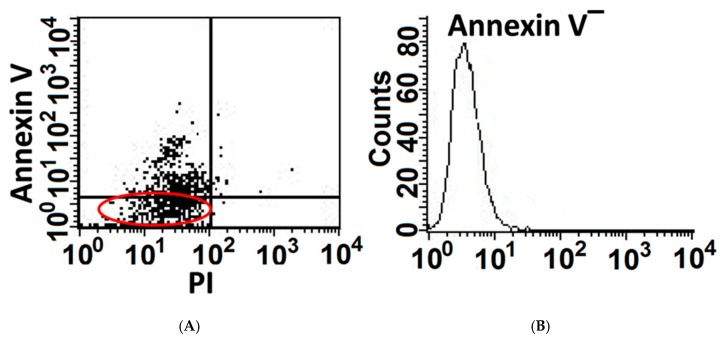
A portion of AECs, mouse lung epithelial (MLE) cells, exposed to bleomycin resist apoptosis and upregulate cell-survival genes. Mouse lung epithelial (MLE12)-cell line following bleomycin (BLM) exposure (200 mU, 1 h). Flow cytometry analysis of Annexin-v staining (**A** Left panel) and following cell sorting of Annexin V-negative cell population (**B** Right panel, Annexin V^(−)^).

We have previously shown that MLEs’ viability decreases and apoptosis increases with growing doses of bleomycin [1]. Here we demonstrate, that despite being exposed to high bleomycin (BLM) concentration (200 mU, 24 h), some MLE cells resist apoptosis (Figure 1A left panel), note annexin-V^(−)^ cells, indicating non-segregated area of the annexin-V in dot-blot.

To further characterize the subset of MLEs resisting cell death, cell sorting by flow cytometry and expansion in culture (HITES) of annexin-V-negative MLEs (BLM-annexin-V^(−)^), was performed (Figure 1A, right panel).

Concomitantly, annexin-V^(−)^ cells showed the upregulation of survival genes which are known to be expressed in response to oxidative-stress and DNA-damage, such as *GADD45*, *P21* and others, but also of genes involved in apoptosis regulation, such as *ATM*, *IAP*’s and *survivin* (Table 1).

### 3.2. BLM-Annexin V^(−)^ AECs Upregulate Ataxia Telangiectasia Mutated (ATM) Protein Expression, Activation and Phosphorylation with other Linked Cell-Survival Proteins, and Proliferate

**Figure 2:**
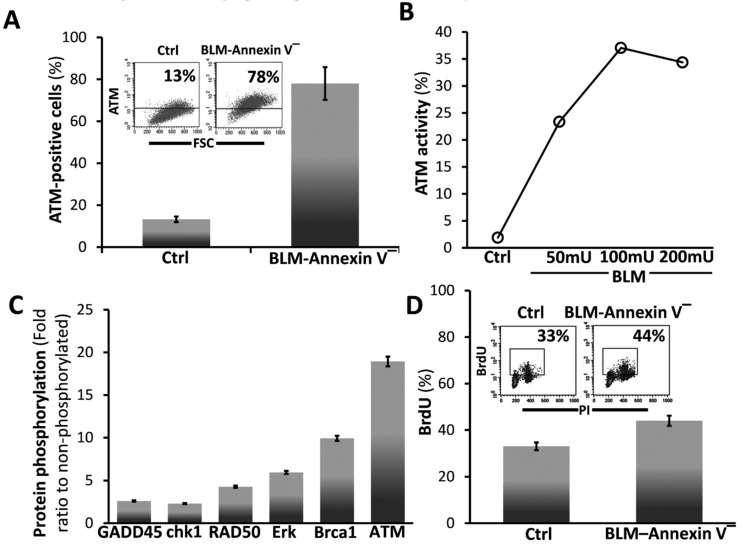
BLM-Annexin V^(−)^ AECs, upregulate ATM protein expression, activation and phosphorylation with other linked cell-survival proteins, and proliferate. (**A**) Flow cytometry analysis (insert) and graphical presentation of ATM expression in Annexin v-negative (BLM-Annexin V^(−)^) MLE cell sorted population vs. control/saline-treaded MLE cells. (**B**) Graphical presentation of ATM activity assay of phosphorylated (ATM-p) in WB of Annexin v-negative following increases BLM concentrations (BLM-Annexin V^(−)^) MLE cell sorted population in 50, 100 and 200 mU of BLM, vs. control/saline-treaded MLE cells. (**C**) Phosphorylation antibody array. Graphical presentation of antibody array incubated with whole-cell lysate from MLE Annexin V(^−^) vs. control/saline-treaded MLE cells. After 1 h of incubation, the membrane was washed with PBS and blotted with HRP-conjugated anti-Ptyr followed by ECL detection. Signaling proteins such as ATM, Erk, RAD50, GADD45, CHK1and Brca-1 are in a phosphorylation state. (**D**) Flow cytometry analysis (inserts) and graphical presentation of BrdU uptake quantified at the S-phase versus PI-total DNA, in MLE Annexin V^(−)^ vs. control/saline-treaded MLE cells.). PI- and PI/annexin V-positive cells were excluded from the presented data. Representative of two experiments, *n* = 4, *p* < 0.02.

ATM is known to function in cell survival [27,28] and to phosphorylate a high number of proteins during double-strand DNA breaks repair. Bleomycin (BLM) induces, ATM in Annexin-V^(−)^ MLEs, (Figure 2A, with graphical presentation, left panel and insert), ATM activation (Figure 2B) and an array of phosphorylated proteins (fold ratio to saline-treated MLEs) of ATM cascade; Erk, ATM, BRCA1, RAD50, GADD45 and chk1 in BLM-Annexin-V^(−)^ MLE cells (Figure 2C). Concomitantly we show here increased proliferation in BrdU uptake from a percentage of 33% in saline-treated MLEs (Ctrl) to 44% (Figure 2D with graphical presentation), in bleomycin-treated MLEs.

### 3.3. BLM-Annexin V^(−)^ AECs Perform β-Catenin Nucleus Translocation and a CD44-Mediated Downregulation of E-Cadherin Expression with N-Cadherin and Vimentin Overexpression

**Figure 3:**
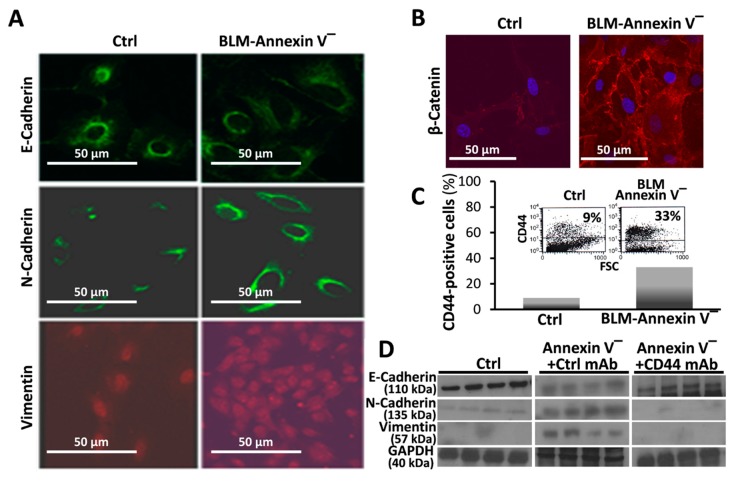
BLM-Annexin V^(−)^ AECs, perform β-catenin nucleus translocation and a CD44-mediated downregulation of E-cadherin expression with N-cadherin and vimentin, overexpression. Annexin v-negative (BLM-Annexin V^(−)^) MLE cell sorted population vs. control/saline-treaded MLE cells. Immunofluorescence of cultured cells in confocal microscope of (**A**) E-Cadherin, N-Cadherin and Vimentin and (**B**), β-catenin showing nucleus translocation, using Rabbit α-β-cathenin (1:100) (Epitomics) and donkey anti-rabbit Cy5 (1:500, Jackson ImmunoResearch, West Grove, PA, USA) as secondary antibody. (**C**) Flow cytometry analysis of CD44 expression (inserts) and graphical presentation using FITC-conjugated KM81 anti-CD44 mAb. (**D**) Western blot of E-cadherin, N-Cadherin and Vimentin assessed in MLE cells (10^6^/mL), pretreated with 0.1 mg/mL blocking KM81 anti-CD44 mAb or isotype-matched control mAb (4D2), (5min, 4 °C). Results are representative of three separate experiments with similar results, *n* = 3–4, *p* < 0.05.

BLM-Annexin-V^(−)^ MLEs upregulate N-cadherin and vimentin with concomitant downregulation of E-cadherin (Figure 3A). Moreover, compared to control MLEs, BLM-Annexin-V^(−)^ MLEs perform β-catenin nucleus translocation (Figure 3B).

CD44 is a known marker of mesenchymal cell phenotype. First we detected, in flow cytometry, increased CD44 expression (Figure 3C, BLM-Annexin-V^(−)^ vs. Ctrl), specifically in BLM-Annexin-V^(−)^ MLEs, compared to control saline-treated MLEs (Figure 3C). EMT was further confirmed in Wb showing decreased E-cadherin with increased N-cadherin and vimentin in BLM-Annexin-V^(−)^ MLE cells compared to controls (Figure 3D, BLM-Annexin-V^(−)^ vs. Ctrl). Moreover, when BLM-Annexin-V^(−)^ MLEs were treated with KM81 mAb (0.1 mg/mL, 5 min, 4 °C), a CD44 antagonist, E-cadherin levels induced by bleomycin, returned significantly to normal (Ctrl, 4D2 mAb) and those of N-cadherin and vimentin, were downregulated, (Figure 3D, BLM-Annexin-V^(−)^ +Ctrl mAb vs. BLM-Annexin-V^(−)^ + CD44 mAb).

### 3.4. Bleomycin-Injured Chimeric-CD44^KO^ Mice Decrease EMT Markers in Their Lungs, and Isolated α-SMA^+^ Cells Decrease their Motility Phenotype In Vitro

**Figure 4:**
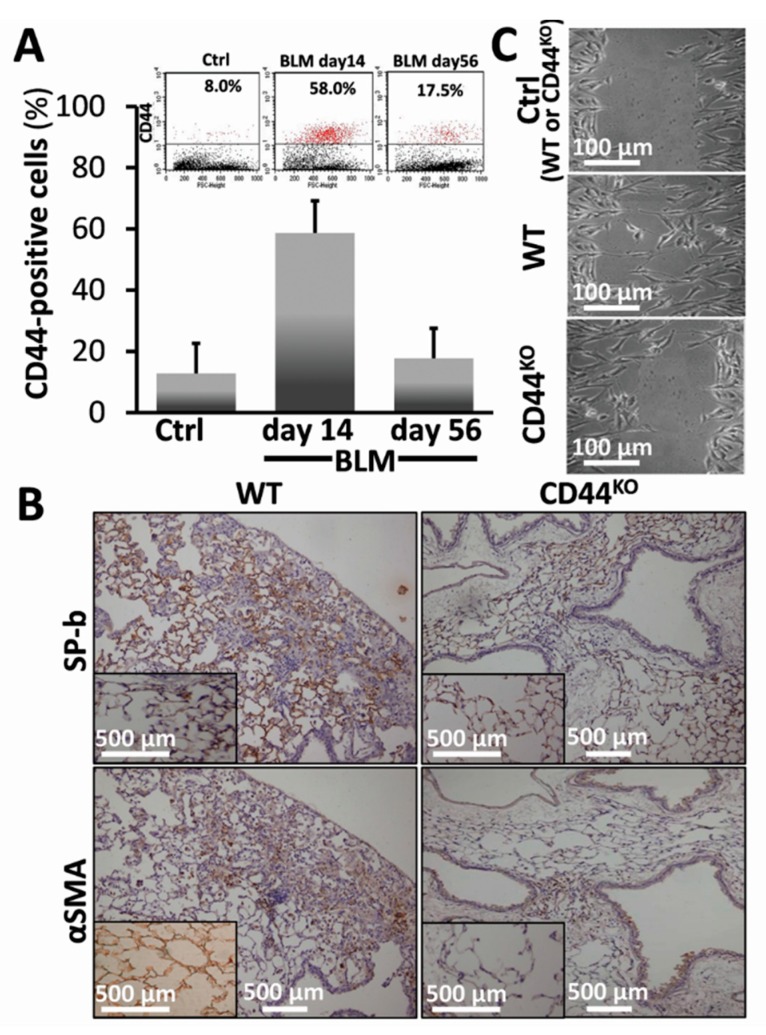
Bleomycin-injured chimeric-CD44^KO^ mice, compared to congenic-controls, decrease EMT markers in their lungs and isolated αSMA-positive cells decrease their motility phenotype in vitro. (**A**) Flow cytometry analysis (inserts) and graphical presentation of CD44 expression in myofibroblasts isolated from C57BL/6 mouse lungs at 14 days (acute fibrosis), and 56 days (tissue regeneration) following bleomycin injury vs. normal-saline control. *p* < 0.05. (**B**) Surfactant-b epithelial cell marker (SP-b) and mesenchymal/myofibroblasts cell marker (αSMA) staining in lung tissue sections from C57BL/6 WT vs. chimeric-CD44^KO^ mice at day 14 of bleomycin and control-saline (inserts). Representative of 4–5 sections in 3 separate experiments with similar results, *n* = 7–10. (**C**) Injury assay of motility. Cells were cultured in medium containing 0.5% BSA and 20% FCS, for 72 h up to 50–70% confluence. The cells were scratched with a pipette tip, washed with PBS. The wounded areas were further cultured in starvation medium 0.5% FCS for additional 24 h. The images of cell recovery and migration were taken under phase contrast microscope. Performed by TIMNA software.

**Figure 5:**
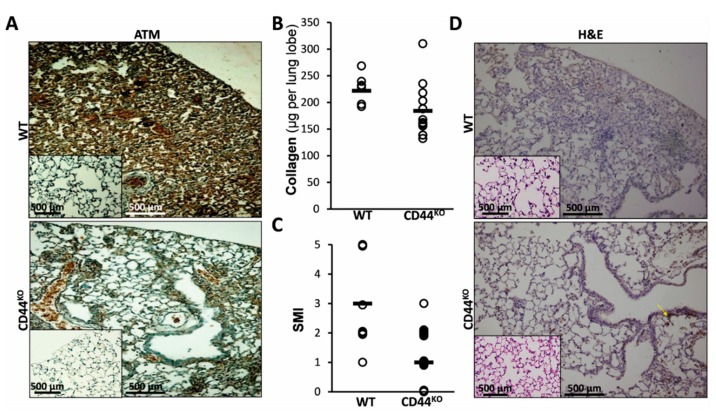
Normal lung regeneration following bleomycin-injury in chimeric-CD44^KO^ mice, compared to congenic-controls. (**A**) ATM staining in lung tissue sections from C57BL/6 WT vs. chimeric-CD44^KO^ mice at day 14 of bleomycin and control-saline (inserts). Representative of 4–5 sections in 3 separate experiments with similar results, *n* = 7–10. (**B**) Graphical presentation of Sircoll-collagen assay and (**C**), of a semi-morphological index (SMI) of H&E staining (see **D**), at day 14 post-BLM in WT and CD44^KO^. Bars (▬) represent median score. Three lung sections were scored from each animal, using a grading scheme as follows: grade 0 – normal tissue; grades 1–5 were used for animals with regenerated tissue (grade 0 to 1) or pulmonary inflammation and fibrosis, with higher grades (2 to 5) indicating more severe lesions [24]. Representative results from three independent experiments. For all comparisons, *p* < 0.05. (**D**) Hematoxylin-eosin (H&E) staining at day 14 post-BLM, in WT and CD44^KO^ murine lung tissue sections. Representative of 4–5 sections in 3 separate experiments with similar results, *n* = 7–10.

We have previously shown [21] that young mice injured with bleomycin (0.03–0.05 mU/mouse) evolve pulmonary fibrosis by day 14 but as opposed to aged mice [10], we show that lung tissue regenerates by day 56 [21]. Here we found CD44 overexpression at fibrosis apex (day 14) in WT C57BL/6 mice compared to control saline-treated mice lung fibroblasts (58% vs. 8%, correspondingly) (Figure 4A, BLM-day 14 vs. Ctrl). CD44 levels returned to almost normal levels (17.5%) at regeneration (Figure 5A, BLM-day 56 vs. 14 and Ctrl; see also graphical presentation (Figure 4A, BLM-day 56 vs. 14 and Ctrl).

Chimeric CD44^KO^ mouse model, with CD44^KO^ mesenchymal cells and WT hematopoietic cells, generated by us as detailed previously [19] allows the assessment of CD44’s role, specifically in mesenchymal cells in tissue regeneration in vivo, thus excluding the possible contribution of CD44-negative immune cells. We exposed chimeric-CD44^KO^ - and control WT- mice to 0.05 mU bleomycin (Figure 4B) or saline (Figure 4B, inserts), and analyzed, at day 14, EMT in IHC, (Figure 4B vs. inserts). SP-b expression was significantly reduced in bleomycin chimeric CD44^KO^ mice (Figure 4B, SP-b in CD44^KO^ vs. WT), compared to controls (Figure 4B, inserts of SP-b in CD44^KO^ vs. WT inserts). Moreover, αSMA^+^ cells, isolated from the lungs of bleomycin-injured chimeric CD44^KO^ mice, compared to WT (day 14), show significantly decreased motility almost equal to those from control saline-treated mice lungs (Ctrl: WT or CD44^KO^), in the “injury model” in vitro (Figure 4C, CD44^KO^ (BLM) vs. WT (BLM)) and (Figure 4C, CD44^KO^ (BLM) vs. WT or CD44^KO^ (Ctrl)).

### 3.5. Normal Lung Regeneration Following Bleomycin-Injury in Chimeric-CD44^KO^ Mice, Compared to Congenic-Controls

ATM, the pro-survival protein, activated in AECs in vitro following bleomycin exposure (Figure 2 and Figure 3) was upregulated in vivo in lungs of bleomycin-injured WT mice (WT in Figure 5A and insert) but downregulated in chimeric CD44^KO^ mice, (Figure 5A, WT vs. CD44^KO^). At 14 days of bleomycin exposure, chimeric CD44^KO^ mice had increased lung tissue regeneration (less fibrosis), as determined using Sircol-red assay by significant decreases in lung collagen deposition (Figure 5B). From an average of 250 microgram per lobe in WT mice lungs to only 110, in chimeric CD44^KO^ mice (Figure 5B, WT vs. CD44^KO^). We used a semi morphological quantitative index (SMI) to grade tissue regeneration vs. fibrosis development (Figure 5C, WT vs. CD44^KO^) in bleomycin-injured mouse lungs (from 1 to a maximum of 5), of H&E-stained sections (Figure 5D, WT vs. CD44^KO^). There was a significant decrease (*p* < 0.05) in the average of SMI grading from 2.6 in WT mice lungs to only 0.5 in mice with CD44^KO^ mesenchymal cells (Figure 5C, WT vs. CD44^KO^). H&E staining show increased regeneration in lung tissue sections of bleomycin- compared to saline-treated chimeric CD44^KO^ mice (Figure 5D, CD44^KO^ vs. WT vs. inserts).

## 4. Discussion

Previously we demonstrated that AECs exposed to bleomycin enter a ROS-mediated stress state and undergo cell death [1]. Here we evaluated AECs survival and lung tissue regeneration vs. lack of regeneration and evolution of fibrosis with a specific aim at ATM, a DNA repair factor, and the role of CD44 in EMT, in vitro, and in vivo, in the murine bleomycin-induced lung injury as an experimental model of ROS-mediated lung injury [10]. Our results focus on a subset of AECs that do not undergo bleomycin apoptosis in vitro (annexin-V^(−)^, Figure 1A) and display an array of survival genes (Figure 1B). ATM expression, and signaling, were significantly increased in AECs/annexin-V^(−)^ subset (Figure 2A–C) and subsequently, their proliferative activity (Figure 2D). Moreover, AECs surviving bleomycin, downregulated epithelial cell marker, E-cadherin and upregulated those of mesenchymal cells, N-cadherin and vimentin (Figure 3A and C) and increased nuclear translocation of β-catenin (Figure 3B) indicating EMT. The proportion of AECs/annexin-V^(−)^, expressing CD44 adhesion molecule was increased (Figure 3C) and KM81, a specific CD44 mAb, decreased markers of EMT in these cells (Figure 3D). Using the bleomycin-induced lung injury in WT mouse lungs, we show, that myofibroblasts isolated from the lungs at times of tissue degradation and lack of regeneration (day 14), increased CD44 expression and decreased it, to normal levels, at times when the pulmonary tissue regenerates (see our previous report on fibrosis kinetics [21] (Figure 4A). Bleomycin- vs. saline-treated WT mice decreased surfactant-b, and increased αSMA- cells in their lungs while chimeric CD44^KO^ mice, with specific knockout mesenchymal cells, show the opposite (Figure 4B). Moreover, myofibroblasts isolated from the lungs of chimeric CD44^KO^ mice, compared to those from WT, lost their migratory capacity (Figure 4C). Concomitantly, these mice show decreased ATM staining in cells of the alveolar interstitium (Figure 5A) with decreased fibrotic pathology (Figure 5B–D), thus evidencing improved regeneration of the lung tissue after bleomycin-induced injury.

The capacity of AECs to undergo EMT under oxidative stress is well established [9,29]. The role CD44 plays in EMT makes it an important target of numerous fields [4,30], including when the tissue fails to regenerate and fibrosis evolves [7,8] and differences in regulation of several known CD44 isoforms, may function differentially and bidirectionally upregulated or downregulated in EMT and, consequently, in fibrosis [8,31]. Our results accordant with others detecting CD44 upregulation in AECs subjected to hypoxia, with corresponding decrease in E-cadherin and increase in vimentin [32]. CD44-mediated signaling in pulmonary fibrosis has been addressed in a number of studies [33,34]. This study corroborated CD44’s role, specifically in mesenchymal cells and in AECs acquiring mesenchymal properties (Figure 3) and in reducing propensity for tissue regeneration following injury. In vivo we found, for the first time that knocking out CD44, specifically in mesenchymal cells, excluding the hematopoietic lineage, leads to significantly improved outcome in the regenerative capacity of the tissue following bleomycin injury, particularly reducing EMT (Figure 4) and pulmonary fibrosis (Figure 5). Moreover, as others [32], we show that myofibroblasts isolated from bleomycin-injured WT mice lungs demonstrated CD44 overexpression and increased motility, while those from chimeric-CD44^KO^, did not (Figure 4). In our model CD44 kinetics in lung myofibroblasts (Figure 4) is in good correlation with BLM-induced pulmonary fibrosis kinetics, which in young mice reaches maximum by day 14–21 and then regenerate the pulmonary tissue by day 28–56 [10]. Although similarly Li et al. showed that fibroblasts isolated from bleomycin-treated CD44-null mice or from WT-mice treated with CD44 mAb, impair invasive capacity, their CD44-null mice are not protected from pulmonary fibrosis and lack tissue regeneration [33]. This could be explained by the fact that Li et al. used CD44-null C57BL/6 mice which does not allow to exclude the contribution of CD44 hematopoietic cells in lung fibrosis [31]. In contrast, we used CD44^KO^ chimeric mice with WT hematopoietic cells.

ATM, involved in stress response, cell cycle regulation, tissue regeneration, apoptosis and survival [35], appears to influence AECs response to injury and fibrosis outcome [36]. We show here (Figure 1, Figure 2 and Figure 3) that apoptosis-resistant AECs undergo adaptation, involving increased ATM expression with phosphorylation of key regulators of cell cycle and survival such as Erk, Brca1, RAD50, GADD45 and chk1, thus barring regeneration of normal lung tissue. Concomitantly, ATM was demonstrated to promote liver fibrosis [37]. Our findings further reveal ATM downregulation in chimeric-CD44^KO^ mice, who attenuate lung fibrosis and thus increase tissue regeneration (Figure 4 and Figure 5). Others, nevertheless, associated ATM deficiency with increased pro-fibrosis cell’s proliferation. For example, myocytes following heart injury [38], pancreatic cells undergoing EMT [39] and, even myofibroblasts in bleomycin-treated ATM-deficient mice [36]. The latter, used ATM-knockout or deficient mice, without excluding hematopoietic-lineage. Moreover, in our study, ATM absence results in regeneration of the lungs of bleomycin-treated mice lacking CD44^+^ myofibroblasts. The role of ATM in fibrosis may also depend on the particular splice variant in question [40] and appears to be strongly depended on the particular condition, tissue, or model investigated [15,36,39,41].

To conclude, our results demonstrate the key role of CD44 in transdifferentiation and motility of AECs surviving stress induced by bleomycin and in subsequent suppression of lung tissue regeneration and promotion of pulmonary fibrosis. This survival of AECs is associated with activation of ATM and ATM-signaling. EMT may represent a possible ATM-mediated cytoprotective mechanism of alveolar epithelial cells under oxidative stress. These results bear further significance for the tackling of the questions of promoting of regenerative capacity and rejuvenation of damaged tissue in aging and aging-associated disease as fibrosis.

## Figures and Tables

**Table 1:** Mouse lung epithelial (MLE) cells exposed to bleomycin upregulate cell-survival genes. Apoptosis superarray membranes spotted with gene specific cDNA. Fragments of 96 apoptosis and survival related genes were with cDNA probes synthesized from three total RNA samples corresponding to cultured Annexin V^(−)^ MLE cell sorted population vs. control/saline-treaded MLE cells (Ctrl). The relative expression levels of the various genes were estimated by comparing signal intensity to *RPLA 13A* housekeeping genes and then quantitated by densitometry after background subtraction. *p* < 0.05, *n* = 3.

Survival Gene	Ctrl	BLM-Annexin V‾
**P53 and ATM**		
*P21*	0	2
*Chk1*	0	3
*Atm*	0	6.9
*Rad 53 (chk 2)*	0.01	2.53
*Hus*	−1	3.16
*Gadd 45*	0	12.6
*Rpa*	4.5	13
*Mdm 2*	8	25
**TNF NF-KB Activation**		
*TRAF 4*	0.2	8.86
*TRAF 3*	1.8	15.19
*TRAF 1*	−1.5	1.9
*I-TRAF*	0.23	5.06
**Regulation of Apoptosis**		
*NAIP 1*	−1	2.53
*Survivin*	11	29
*Bik*	B3	1.9
*Bim*	−2	3.8
*Apaf-1*	−0.4	1.9

## References

[1] Wallach-Dayan S.B., Izbicki G., Cohen P.Y., Gerstl-Golan R., Fine A., Breuer R. (2006). Bleomycin initiates apoptosis of lung epithelial cells by ROS but not by Fas/FasL pathway. Am. J. Physiol. Lung Cell Mol. Physiol..

[2] Nicolás F.J., Lehmann K., Warne P.H., Hill C.S., Downward J. (2003). Epithelial to Mesenchymal Transition in Madin-Darby Canine Kidney Cells Is Accompanied by Down-regulation of Smad3 Expression, Leading to Resistance to Transforming Growth Factor-β-induced Growth Arrest. J. Biol. Chem..

[3] Thiery J.P., Acloque H., Huang R.Y.J., Nieto M.A. (2009). Epithelial-mesenchymal transitions in development and disease. Cell.

[4] Iwatsuki M., Mimori K., Yokobori T., Ishi H., Beppu T., Nakamori S., Baba H., Mori M. (2010). Epithelial–mesenchymal transition in cancer development and its clinical significance. Cancer Sci..

[5] Thannickal V.J., Toews G.B., White E.S., Lynch J.P., Martinez F.J. (2004). Mechanisms of pulmonary fibrosis. Annu. Rev. Med..

[6] Misra S., Hascall V.C., Markwald R.R., Ghatak S. (2015). Interactions between Hyaluronan and Its Receptors (CD44, RHAMM) Regulate the Activities of Inflammation and Cancer. Front. Immunol..

[7] Chang S.-H., Yeh Y.-H., Lee J.-L., Hsu Y.-J., Kuo C.-T., Chen W.-J. (2017). Transforming growth factor-β-mediated CD44/STAT3 signaling contributes to the development of atrial fibrosis and fibrillation. Basic Res. Cardiol..

[8] Rampanelli E., Rouschop K.M.A., Claessen N., Teske G.J.D., Pals S.T., Leemans J.C., Florquin S. (2014). Opposite role of CD44-standard and CD44-variant-3 in tubular injury and development of renal fibrosis during chronic obstructive nephropathy. Kidney Int..

[9] Sakuma Y. (2017). Epithelial-to-mesenchymal transition and its role in EGFR-mutant lung adenocarcinoma and idiopathic pulmonary fibrosis. Pathol. Int..

[10] Tashiro J., Rubio G.A., Limper A.H., Williams K., Elliot S.J., Ninou I., Aidinis V., Tzouvelekis A., Glassberg M.K. (2017). Exploring Animal Models That Resemble Idiopathic Pulmonary Fibrosis. Front. Med. (Lausanne).

[11] Kinnula V.L., Fattman C.L., Tan R.J., Oury T.D. (2005). Oxidative Stress in Pulmonary Fibrosis. Am. J. Respir. Crit. Care Med..

[12] Bernstein C., Bernstein H., Payne C.M., Garewal H. (2002). DNA repair/pro-apoptotic dual-role proteins in five major DNA repair pathways: Fail-safe protection against carcinogenesis. Mutat. Res..

[13] Valerie K., Povirk L.F. (2003). Regulation and mechanisms of mammalian double-strand break repair. Oncogene.

[14] Dent P., Yacoub A., Contessa J., Caron R., Amorino G., Valerie K., Hagan M.P., Grant S., Schmidt-Ullrich R. (2003). Stress and radiation-induced activation of multiple intracellular signaling pathways. Radiat. Res..

[15] Tang S., Hou Y., Zhang H., Tu G., Yang L., Sun Y., Lang L., Tang X., Du Y., Zhou M. (2015). Oxidized ATM promotes abnormal proliferation of breast CAFs through maintaining intracellular redox homeostasis and activating the PI3K-AKT, MEK-ERK, and Wnt-β-catenin signaling pathways. Cell Cycle.

[16] Stagni V., Manni I., Oropallo V., Mottolese M., Di Benedetto A., Piaggio G., Falcioni R., Giaccari D., Di Carlo S., Sperati F. (2015). ATM kinase sustains HER2 tumorigenicity in breast cancer. Nat. Commun..

[17] Schmits R., Filmus J., Gerwin N., Senaldi G., Kiefer F., Kundig T., Wakeham A., Shahinian A., Catzavelos C., Rak J. (1997). CD44 regulates hematopoietic progenitor distribution, granuloma formation, and tumorigenicity. Blood.

[18] Chen D., McKallip R.J., Zeytun A., Do Y., Lombard C., Robertson J.L., Mak T.W., Nagarkatti P.S., Nagarkatti M. (2001). CD44-deficient mice exhibit enhanced hepatitis after concanavalin A injection: Evidence for involvement of CD44 in activation-induced cell death. J. Immunol..

[19] Golan-Gerstl R., Wallach-Dayan S.B., Amir G., Breuer R. (2007). Epithelial Cell Apoptosis by Fas Ligand–Positive Myofibroblasts in Lung Fibrosis. Am. J. Respir. Cell Mol. Biol..

[20] Egger C., Cannet C., Gérard C., Jarman E., Jarai G., Feige A., Suply T., Micard A., Dunbar A., Tigani B. (2013). Administration of bleomycin via the oropharyngeal aspiration route leads to sustained lung fibrosis in mice and rats as quantified by UTE-MRI and histology. PLoS ONE.

[21] Wallach-Dayan S.B., Elkayam L., Golan-Gerstl R., Konikov J., Zisman P., Dayan M.R., Arish N., Breuer R. (2015). Cutting edge: FasL(+) immune cells promote resolution of fibrosis. J. Autoimmun..

[22] Wallach-Dayan S.B., Golan-Gerstl R., Breuer R. (2007). Evasion of myofibroblasts from immune surveillance: A mechanism for tissue fibrosis. Proc. Natl. Acad. Sci. USA.

[23] Cohen P.Y., Breuer R., Wallach-Dayan S.B. (2018). A Profibrotic Phenotype in Naïve and in Fibrotic Lung Myofibroblasts Is Governed by Modulations in Thy-1 Expression and Activation. Mediators Inflamm..

[24] Golan-Gerstl R., Wallach-Dayan S.B., Zisman P., Cardoso W.V., Goldstein R.H., Breuer R. (2012). Cellular FLICE-like inhibitory protein deviates myofibroblast fas-induced apoptosis toward proliferation during lung fibrosis. Am. J. Respir. Cell Mol. Biol..

[25] Cohen P.Y., Breuer R., Wallach-Dayan S.B. (2009). Thy1 up-regulates FasL expression in lung myofibroblasts via Src family kinases. Am. J. Respir. Cell Mol. Biol..

[26] Qian M., Liu Z., Peng L., Tang X., Meng F., Ao Y., Zhou M., Wang M., Cao X., Qin B. (2018). Boosting ATM activity alleviates aging and extends lifespan in a mouse model of progeria. Elife.

[27] Clevers H. (2006). Wnt/β-Catenin Signaling in Development and Disease. Cell.

[28] Logan C.Y., Nusse R. (2004). The Wnt signaling pathway in development and disease. Annu. Rev. Cell Dev. Biol..

[29] Salton F., Volpe M.C., Confalonieri M. (2019). Epithelial^−^Mesenchymal Transition in the Pathogenesis of Idiopathic Pulmonary Fibrosis. Medicina (Kaunas).

[30] Leng Y., Abdullah A., Wendt M.K., Calve S. (2019). Hyaluronic acid, CD44 and RHAMM regulate myoblast behavior during embryogenesis. Matrix Biol..

[31] Jordan A.R., Racine R.R., Hennig M.J.P., Lokeshwar V.B. (2015). The Role of CD44 in Disease Pathophysiology and Targeted Treatment. Front. Immunol..

[32] Guo L., Xu J., Liu L., Liu S., Zhu R. (2015). Hypoxia-Induced Epithelial-Mesenchymal Transition Is Involved in Bleomycin-Induced Lung Fibrosis. BioMed Res. Int..

[33] Li Y., Jiang D., Liang J., Meltzer E.B., Gray A., Miura R., Wogensen L., Yamaguchi Y., Noble P.W. (2011). Severe lung fibrosis requires an invasive fibroblast phenotype regulated by hyaluronan and CD44. J. Exp. Med..

[34] Kasper M., Bierhaus A., Whyte A., Binns R.M., Schuh D., Müller M. (1996). Expression of CD44 isoforms during bleomycin-or radiation-induced pulmonary fibrosis in rats and mini-pigs. Histochem. Cell Biol..

[35] Shiloh Y., Ziv Y. (2013). The ATM protein kinase: Regulating the cellular response to genotoxic stress, and more. Nat. Rev. Mol. Cell Biol..

[36] Duecker R., Baer P., Eickmeier O., Strecker M., Kurz J., Schaible A., Henrich D., Zielen S., Schubert R. (2018). Oxidative stress-driven pulmonary inflammation and fibrosis in a mouse model of human ataxia-telangiectasia. Redox Biol..

[37] Daugherity E.K., Balmus G., Al Saei A., Moore E.S., Abi Abdallah D., Rogers A.B., Weiss R.S., Maurer K.J. (2012). The DNA damage checkpoint protein ATM promotes hepatocellular apoptosis and fibrosis in a mouse model of non-alcoholic fatty liver disease. Cell Cycle.

[38] Foster C.R., Zha Q., Daniel L.L., Singh M., Singh K. (2012). Lack of ATM induces structural and functional changes in the heart: Role in β-adrenergic receptor-stimulated apoptosis. Exp. Physiol..

[39] Russell R., Perkhofer L., Liebau S., Lin Q., Lechel A., Feld F.M., Hessmann E., Gaedcke J., Güthle M., Zenke M. (2015). Loss of ATM accelerates pancreatic cancer formation and epithelial–mesenchymal transition. Nat. Commun..

[40] Andreassen C.N., Overgaard J., Alsner J., Overgaard M., Herskind C., Cesaretti J.A., Atencio D.P., Green S., Formenti S.C., Stock R.G. (2006). ATM sequence variants and risk of radiation-induced subcutaneous fibrosis after postmastectomy radiotherapy. Int. J. Radiat. Oncol. Biol. Phys..

[41] Chen W.-T., Ebelt N.D., Stracker T.H., Xhemalce B., Van Den Berg C.L., Miller K.M. (2015). ATM regulation of IL-8 links oxidative stress to cancer cell migration and invasion. Elife.

